# The impact of Host vs. Graft mismatches on rejection of haploidentical bone marrow transplants in thalassemia patients using posttransplant cyclophosphamide

**DOI:** 10.1038/s41409-019-0692-0

**Published:** 2019-09-30

**Authors:** Priya Marwah, Rajpreet Soni, Stalin Ramprakash, C. P. Raghuram, Deepa Trivedi, Rajat Kumar Agarwal, Rakesh Dhanya, Amit Sedai, Ankita Kumari, Lalith Parmar, Lawrence Faulkner

**Affiliations:** 1 South East Asia Institute for Thalassemia, Jaipur, India; 2 People Tree Hospitals, Bangalore, India; 3 Care Institute for Medical Sciences, Ahmedabad, India; 4 Sankalp India Foundation, Bangalore, India; 5 Cure2Children Foundation, Florence, Italy

**Keywords:** Risk factors, Genetics research

## To the Editor:

Blood or marrow transplantation can cure patients with transfusion-dependent thalassemia (TDT) [1] and normalize their long-term health-related quality of life [2]. In regions where TDT is most prevalent, 38–60% of transplant candidates may find a fully matched-related donor [3], thus a substantial proportion of patients require alternative donors. The use of posttransplant cyclophosphamide (PTCy) as well as ex vivo T-cell depletion methods have allowed to safely perform transplants across HLA barriers [4–6]. Because of its simplicity and inexpensiveness, PTCy seems a particularly attractive option for centers in lower-middle-income countries (LMIC) in South-East Asia where TDT is the most frequent life-threatening noncommunicable disorder of childhood and a major financial burden to families and health care systems [7]. Outcomes using partially matched-related donors (PMRD) with the PTCy approach have been shown to be comparable with those using unrelated or related fully matched donors [5, 8], but unrelated donors in LMIC are often unavailable and/or unaffordable. In the PMRD transplantation context the relevance of donor-specific antibodies (DSA) is well established [9], less so that of different degrees and types of HLA mismatches which, in fact, are not generally considered relevant [10].

We retrospectively assessed the impact of DSA as well as that of Host vs. Graft (HVG) set ups, i.e., when the recipient is homozygous for one or more HLA specificities while the donor is not, so that for those HLA specificities the recipient has the potential to react towards the unshared allele of the donor but not vice versa. A total of 28 consecutive partially matched-related BMTs where analyzed, these were performed between March 2017 and December 2018 in three centers in India: The South-East Asia Institute for Thalassemia in Jaipur, Rajasthan (20 cases), the People Tree Hospitals in Bangalore, Karnataka (seven cases), and the Care Institute for Medical Sciences in Ahmedabad (one case). Selection criteria included a diagnosis of TDT, lack of a fully matched-related donor, age at BMT <15 years (range 1.5–13.5 years, median 5.3), no significant hepatosplenomegaly (<2 cm from costal margin) or serum ferritin >5000 ng/mL pre-BMT. None of the patients underwent a liver biopsy and thus would be considered Pesaro class I–II given the absence of hepatomegaly [1]. A common online electronic medical record system and collaboration platform where data were entered prospectively on a daily basis (BMTPlus^®^, Jagrity Innovations, Bangalore, India, www.bmtplus.net) [11], and a single transplant approach approved by centers’ IRB was used. The preparative regime, modified from Anurathapan at al. [5], is outlined in Supplementary Fig. 1. All caretakers provided informed consent to share personal data as well as for the BMT procedure. All patients received G-CSF-primed (5 µg/kg twice daily from day −5 to −1) bone marrow with a total nucleated cell dose ranging from 12.1 to 52.9 × 10^8^/recipient kg (median 16.3). Post-BMT all blood products were irradiated with ≥25 Gy. Autologous marrow was cryopreserved in all cases. Chimerism was monitored at least at 1, 2, 4, and 8 months by molecular (STR) analysis or Y chromosome cytogenetics or fluorescent in-situ hybridization when informative. All patients and immediate family members were HLA-typed by sequence-based high-resolution typing confirmed in two independent samples in different laboratories. All but one patient were evaluated for DSA status.

Data were collected and analyzed on 31 May 2019. Fisher’s exact tests were used to compare proportions and Mann–Whitney nonparametric test were used to compare continuous distribution values. Kaplan–Meier survival curves were compared using the log-rank (Mantel–Cox) test. All *P* values are two-tailed. Statistical analysis was performed using GraphPad Prism version 5.00 for Windows, GraphPad Software, San Diego, California, USA, www.graphpad.com.

Six out of twenty-five patients (24%) had a rejection, with no significant differences in terms of sex, maternal vs. paternal donor, or cell dose between patient who rejected and those who did not (see Table 1). A total of 4 patients out of 27 evaluated for DSA where positive with mean fluorescent intensity >2000, 2/6 in the rejection group (33%) and 2/21 in the nonrejection one (9%). In the rejection group 3/6 (50%) had a HVG set up (patient characteristics are summarized in Table 2). Actuarial rejection proportion went from 5% in patients with neither DSA-positivity nor HVG set up, to 56% in those with either one, see Supplementary Fig. 2. There was no overlap between DSA-positive patients and those with HVG set up. Among patients who did not reject 4/22 (18%) had a HVG set up, all with unilateral homozygosities at the A locus, in 2 the donor was DRB1 and DQB1 compatible, in 1 was also B and C compatible, and in 1 was DRB1 and DQB1 unilaterally homozygous, thus there was a concomitant HVG and GHV set up. Both DSA-positive patients who did not reject had a GVH set up. None of the patients who rejected had a GVH set up. Of the 21 thalassemia-free patients, 18 (86%) have >95% donor chimerism, 2/22 (9%) of engrafted patients developed grade III or IV GHVD, no case of extensive chronic GHVD has been observed so far. One patient died of grade IV GVHD and had a GVH set up, otherwise there was no apparent correlation between GVH set up and actual occurrence of GVHD. At a median follow up of 13 months (range 5.7–26.4) transplant-related mortality was 24% vs. 17% in DSA^+^ HVG^+^ and DSA^−^ HVG^−^ patients respectively with a *P* value of 0.53.

**Table 1:** Data summary of patients who rejected vs. those who did not

	Patients who rejected	Patients who did not reject	*P*
Total patients	6	22	
Median age (range)	6.5 (3.4–10)	4.4 (1.5–13.5)	0.40
Sex	4 males, 2 females	16 males, 6 females	1
Donor	5 mother, 1 father	13 mother, 6 father	1
Marrow cell dose ×10^8^/kg	17.3 (16–21.2)	16.2 (12.1–52.9)	0.52
Marrow white cell count/µL	55,445 (31,730–75,600	68,215 (34,790–252,100)	0.15

**Table 2:** Patients characteristics

UPN	Age at BMT	Sex	Donor	FU	Pre-BMT Blood Tx	Cell dose (×10^8^/kg)	Day to ANC 500	Day to plt 20,000	DSA	HLA notes	DSA + or HVG +	Latest donor chimerism	Rej	Acute GVHD	CMV activation	Status	Notes
INA16034	4.3	F	Mother	26.4	79	23	17	16	Neg	A compatible	No	100	No	2	Yes	A&TF	
INA15054	2.2	M	Father	25.0	28	15.38	17	15	Neg	A compatible	No	61	No	0	Yes	A&TF	
INA15007	3.9	F	Mother	17.2	69	18.5	14	12	Not done	B & DPB1 compatible	No	100	No	1	No	A&TF	
INA15020	3.4	M	Mother	18.4	57	21.7	24	15	Neg		No	99	No	2	No	A&TF	
INA12018	8.9	M	Mother	13.7	120	15.4	19	20	Neg	DRB1, DQB1 & DPB1 compatible	No	100	No	0	No	A&TF	Possible IFD
INA13107	8	M	Mother	16.2	90	20.5	26	14	Neg	DQB1 GVH vector	No	100	No	0	No	A&TF	MAS
INA12057	9.4	M	Mother	NA	201	16.05	26	35	Neg	DRB1, DQB1 & DPB1 compatible	No	100	No	0	No	TRM	BK virus HC, ICH
INA17022	5.1	F	Mother	13.0	50	13.7	24	18	Neg		No	100	No	1	No	A&TF	
INE16041	5.9	F	Mother	12.2	117	52.9	18	18	Neg	B, C, DRB1 & DQB1 GVH vector	No	97	No	0	No	A&TF	
INE17035	13.5	M	Mother	NA	147	12.14	20	25	Neg	DRB1, DQB1 & DPB1 compatible	No	97	No	0	No	TRM	Probabble IFD
INA10055	11.5	M	Father	11.2	198	13.28	24	27	Neg	DRB1, DQB1 & DPB1 compatble, A HVG vector	No	100	No	2	No	A&TF	
INA17041	2	M	Mother	10.3	20	14.12	13	16	Neg	A, C, DRB1, DQB1 & DPB1 compatible	No	100	No	2	No	A&TF	
INE15024	3	M	Mother	9.5	36	44.4	14	18	Neg	DRB1 compatible, DQB1 GVH vector	No	100	No	0	No	A&TF	
AFC17001	3.4	M	Mother	8.5	57	18.4	NA	NA	Neg	DPB1 compatible	No	0	d + 30	0	No	A&T	PGF-PP, Probable IFD
INA11012	9.5	F	Mother	7.5	204	16	17	39	Neg	C compatible	No	100	No	0	No	A&TF	2nd BMT after MRD rejection, MAS, SOS
INA13015	10.7	M	Father	6.5	232	16	18	19	Neg		No	100	No	2	No	A&TF	
INA13128	7	M	Father	6.1	144	14.3	20	16	Neg	DRB1 & DQB1 GVH vector	No	98	No	0	No	A&TF	
INM17025	3.9	M	Mother	5.7	69	23.8	18	19	Neg	B, C, DRB1 & DQB1 compatible, A HVG vector	No	84	No	0	No	A&TF	Responded to DLI for EMC
INA17009	2.1	M	Father	NA	12	16.26	19	18	Neg	A, C, DPB1 GVH vector	No	NA	No	4	No	TRM	Severe GVHD-Sepsis
INA14043	3.5	M	Father	10.9	60	19.13	23	16	Pos	DRB1 & DQB! GVH vector, A HVG vector	Yes	100	No	2	Yes	Dead	Death unrelated to BMT
INA16078	5.8	M	Mother	18.9	115	16.3	NA	NA	Neg	C compatible, DRB1 HVG vector	Yes	0	d + 29	0	No	A&T	PGF-EAR
INE16076	4.2	F	Mother	18.3	76	14.6	21	29	Neg	DRB1 & QB1 compatible, A HVG vector	Yes	100	No	0	Yes	A&TF	
INA16041	5.5	M	Mother	NA	108	20.16	21	19	Pos	C compatible	Yes	3	d + 43	0	No	TRM	SGF
INC15040	8.3	F	Mother	14.2	175	16	NA	NA	Pos		Yes	0	d + 25	0	Yes	A&T	PGF-PP, Provem IFD
INA17120	1.5	M	Mother	14.1	12	18.9	20	36	Pos	A GVH vector	Yes	100	No	1	No	A&TF	
INE17044	10	F	Father	13.0	170	21.21	35	54	Neg	DRB1 & DPB1 HVG vector	Yes	0	d + 20	0	No	A&T	PGF-PP, SOS
INA17021	7.3	M	Mother	11.8	90	16.21	NA	NA	Neg	A HVG vector	Yes	0	d+25	0	No	A&T	PGF-PP
INA15003	4.6	M	Mother	NA	86	17.4	NA	NA	Neg	A HVG vector	Yes	100	No	3	No	TRM	MAS

*FU* follow-up, *Rej* rejection, *A&TF* alive and thalassemia-free, *A&T* alive with thalassemia, *TRM* transplant-related mortality, *MAS* macrophage activation syndrome, *HC* hemorrhagic cystitis, *ICH* intracranial hemorrhage, *IFD* invasive fungal disease, *PGF* primary graft failure, *PP* persistent pancytopenia, *SOS* synusoidal obstructive syndrome, *EMC* early mixed chimerism (<day +60), *EAR* early autologous reconstitution, *SGF* secondary graft failure

Thalassemia seems an ideal model to study the role of immunogenetic factors in the HVG direction because of its homogeneity, functional immune system and exposure to multiple transfusions resulting in higher potential for rejection compared with the hematological malignancy context. The observation that HLA HVG disparities can affect rejection in thalassemia patients has been previously reported in the unrelated setting [12] but not in the haploidentical-related one. A factor which might have contributed to not having identified this HLA vector effect previously in haploidentical BMT is that high-resolution typing is not routinely employed for related donor identification [13], while high-resolution mismatches may have the same clinical significance as low-resolution ones [14]. In fact, the identification of a HLA vector is based on the assumption of true homozygosity of one or more HLA alleles.

We believe that even if PTCy is quite effective in inducing tolerance, some degree of escape still remains since GHVD is not infrequent albeit generally mild and manageable. The same maybe true in the HVG (rejection) direction.

In conclusion, these findings may have important practical implications for the selection of partially matched donors for nonmalignant conditions in which rejection is a potential issue. With all the limitations of a small case series, in our experience the presence of a HVG set up in the context of thalassemia seems as impactful on rejection as that of DSA. The potential implication is that in this context it might be advisable to get high-resolution HLA typing and possibly consider the use of unrelated donors in the presence of unilateral recipient’s HLA homozygosities. This occurrence maybe more frequent in populations with high consanguinity or close ethnicity, like in the Indian subcontinent. The impact of HLA vectors in the HVG or GVH direction in haploidentical transplantation for thalassemia may deserve to be assessed in larger studies.

## Supplementary information

Supplemental Fig 1

Supplemental Fig 2
