# Supplementary figures and images for: The impact of Host vs. Graft mismatches on rejection of haploidentical bone marrow transplants in thalassemia patients using posttransplant cyclophosphamide
Source: Bone Marrow Transplant. 2019 Sep 30;55(9):1824–8. doi: 10.1038/s41409-019-0692-0 (PMC7452814; doi:10.1038/s41409-019-0692-0)

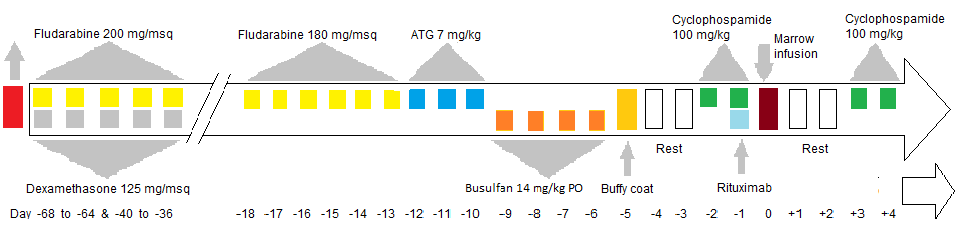

Supplement: Supplementary file 1 — Supplemental Fig 1 [file 41409_2019_692_MOESM1_ESM.png]

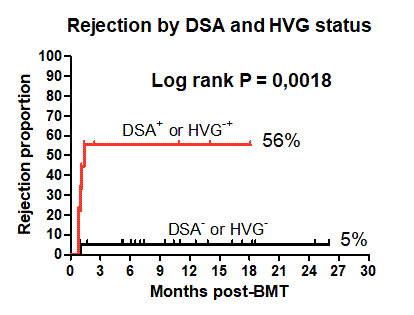

Supplement: Supplementary file 2 — Supplemental Fig 2 [file 41409_2019_692_MOESM2_ESM.png]
